# Biofilm-*i*: A Platform for Predicting Biofilm Inhibitors Using Quantitative Structure—Relationship (QSAR) Based Regression Models to Curb Antibiotic Resistance

**DOI:** 10.3390/molecules27154861

**Published:** 2022-07-29

**Authors:** Akanksha Rajput, Kailash T. Bhamare, Anamika Thakur, Manoj Kumar

**Affiliations:** 1 Virology Unit and Bioinformatics Centre, Institute of Microbial Technology, Council of Scientific and Industrial Research (CSIR), Sector 39-A, Chandigarh 160036, India; akanksharajput.bio@gmail.com (A.R.); kailash@imtech.res.in (K.T.B.); anamikathakur@imtech.res.in (A.T.); 2 Academy of Scientific and Innovative Research (AcSIR), Ghaziabad 201002, India

**Keywords:** antibiotic drug resistance, biofilm, inhibitors, chemical descriptors, QSAR, predictor

## Abstract

Antibiotic drug resistance has emerged as a major public health threat globally. One of the leading causes of drug resistance is the colonization of microorganisms in biofilm mode. Hence, there is an urgent need to design novel and highly effective biofilm inhibitors that can work either synergistically with antibiotics or individually. Therefore, we have developed a recursive regression-based platform “Biofilm-*i*” employing a quantitative structure–activity relationship approach for making generalized predictions, along with group and species-specific predictions of biofilm inhibition efficiency of chemical(s). The platform encompasses eight predictors, three analysis tools, and data visualization modules. The experimentally validated biofilm inhibitors for model development were retrieved from the “aBiofilm” resource and processed using a 10-fold cross-validation approach using the support vector machine and andom forest machine learning techniques. The data was further sub-divided into training/testing and independent validation sets. From training/testing data sets the Pearson’s correlation coefficient of overall chemicals, Gram-positive bacteria, Gram-negative bacteria, fungus, *Pseudomonas aeruginosa*, *Staphylococcus aureus*, *Candida albicans,* and *Escherichia coli* was 0.60, 0.77, 0.62, 0.77, 0.73, 0.83, 0.70, and 0.71 respectively via Support Vector Machine. Further, all the QSAR models performed equally well on independent validation data sets. Additionally, we also checked the performance of the random forest machine learning technique for the above datasets. The integrated analysis tools can convert the chemical structure into different formats, search for a similar chemical in the aBiofilm database and design the analogs. Moreover, the data visualization modules check the distribution of experimentally validated biofilm inhibitors according to their common scaffolds. The Biofilm-*i* platform would be of immense help to researchers engaged in designing highly efficacious biofilm inhibitors for tackling the menace of antibiotic drug resistance.

## 1. Introduction

Biofilms are highly differentiated conglomerate masses of microbes that are enclosed in an extracellular polymeric substance (EPS) matrix [1]. Planktonic bacteria undergo numerous changes to transform into biofilms [2]. Various stages of biofilm include attachment, proliferation, maturation, and dispersion. Initially, the planktonic bacteria begin colonization by adsorbing to any surface through reversible followed by irreversible forces. Next, proliferation starts through multiple cell divisions preceded by their maturation through numerous physiological changes such as oxygen gradient, efflux pumps, division of labor, etc. Finally, dispersal and colonization of the new substratum occur via various factors e.g., enzymes, shear stress, and many more [3,4,5]. Despite various factors, quorum sensing (QS), a cell-to-cell communication [6] among microbes, is considered a major cause of switching from the planktonic form to biofilm mode [7,8]. Moreover, QS is also reported within the biofilm and is a major factor in strengthening biofilms [7]. The interconnection between QS and biofilms was termed sociomicrobiology by Greenberg et al. [9]. However, biofilms are beneficial to microbes, which in turn, is a serious concern for mankind [1].

The city of microbes i.e., biofilm, causes various severe health consequences to humans by significantly protecting microbes from antibiotics, macrophages, shear stress, etc. [10]. In biofilm mode, the bacteria are known to become 10–1000-fold more resistant to antibiotics [11]. There are various mechanisms by which the biofilms become antibiotics resistant namely, slower penetration of antibiotics, the emergence of a zone of slow growing or non-growing bacteria, expression of the adaptive stress response by some cells, differentiation of a few cells as highly protected persisters, antibiotics-induced expression of efflux pumps, protection by the EPS matrix, etc. [12,13]. According to the World Health Organization (Geneva, Switzerland), antibiotic resistance is considered one of the biggest threats globally. Therefore, various strategies have been designed to target biofilms (the major cause of antibiotic resistance). A promising approach is the development of biofilm inhibitors, which can be used either synergistically with antibiotics or alone to tackle antibiotics resistance [12,14,15,16].

Numerous biofilm inhibitors have been designed in the last three decades to degrade the biofilms with diverse natures and modes of action [15,16]. They are (phyto)chemicals, peptides, nanoparticles, biosurfactants, bacterial or fungal or algal abstracts, enzymes, antibodies, phages, and many more [16,17,18]. Biofilm inhibitors are designed to target the biofilms in innumerable ways such as matrix components, disrupting the QS within biofilms, adhesion, cell division, etc. [15]. These inhibitors are natural and (semi)synthetic and designed to work against bacteria (Gram-positive and Gram-negative) and fungus or yeast. The biofilm inhibitors have been proven to be a boon towards the global threat of antibiotic resistance against both ESKAPE [16] and non-ESKAPE pathogens, *Staphylococcus aureus* [17], *Pseudomonas aeruginosa* [19], *Staphylococcus epidermidis* [20], and *Acinetobacter baumannii* [14]. Hence, there is a need to design novel and more effective biofilm inhibitors to fight against recalcitrant biofilms on medical devices, inside the human body, water supplies, fermenters, etc.

The development of bioinformatics tools would be of great help in speeding up the research in the field. In this regard, we developed the first comprehensive repository for anti-biofilm agents termed “aBiofilm” with a total of 5027 entries over three decades [15]. A few methods are available in the literature to predict the biofilm inhibition efficacy of peptides and chemicals, but they adopted different approaches than our current study. For example, for predicting the anti-biofilm peptides, the dPABBs method was developed using a classification-based approach [21]; Gupta et al., developed a classification-based method to predict the biofilm inhibiting peptides [22]; the BIPEP method is a sequence-based predictor for identifying the inhibition efficiency of peptides [23]. However, in the case of chemicals, only two methods are available, based on a classification approach, namely the aBiofilm predictor developed by our group using experimentally validated data [15] and the Molib predictor developed using the data from public repositories such as KEGG [24]. Therefore, to fine-tune the biofilm inhibition efficacy of molecules, we developed the “biofilm-*i*” method using a recursive regression-based approach on experimentally validated molecules using their percentage inhibition taken from the aBiofilm database [15]. The current study includes the first quantitative structure–activity relationship (QSAR) based prediction algorithm named “biofilm-*i*” to predict the anti-biofilm potential of chemicals. The current algorithm can predict the biofilm inhibition efficiency of chemicals in regards to different categories namely, overall generalized chemicals as well as some specific species e.g., *Staphylococcus aureus* (Gram-positive bacteria), *Pseudomonas aeruginosa* (Gram-negative bacteria), *Candida albicans* (fungus or yeast), and *Escherichia coli* (Gram-negative bacteria).

## 2. Material and Methods

### 2.1. Data Collection

The prediction algorithm for identifying the chemicals targeting the biofilm was developed using highly curated data from the comprehensive aBiofilm resource [15]. The quality control was performed in the following steps:

1. Initially, for making the generalized predictor, we extracted 884 unique chemicals with biofilm inhibition potential that varies from 0–100%.
2. For the group-specific predictors, 384, 498, and 158 chemicals were retrieved for Gram-positive, Gram-negative bacteria, and fungus, respectively.
3. For the species-specific algorithms, we selected organisms with a number of non-redundant biofilm inhibitors >100. Thus, we identified four organisms: Staphylococcus aureus (Gram-positive bacteria), Pseudomonas aeruginosa (Gram-negative bacteria), Candida albicans (fungus or yeast), and Escherichia coli (Gram-negative bacteria). *S. aureus*, *P. aeruginosa*, *C. albicans*, and *E. coli* possess 239, 301, 152 and 103 biofilm inhibiting chemicals, respectively.

### 2.2. Quantitative Structure–Activity Relationship (QSAR) Based Model Development

QSAR is used to establish the relationship between biological activity and the physicochemical properties of a category of molecules [25]. Therefore, we used the QSAR approach in this study for two important processes. Firstly, the development of the QSAR model so it is able to describe the relationship between chemical structures and the biological activity of a set of compounds. Secondly, the developed model is used for the prediction of activities of new compounds [26]. However, the initial step of model development includes the division of complete datasets into training/testing and independent validation data sets. Further, the training data set is used for model development and the validation dataset is used for cross-checking the developed model [27].

All the datasets were further subdivided into training/testing (T) and independent validation (V) data sets. For generalized chemicals, Gram-positive bacteria, Gram-negative bacteria, fungus, *S. aureus*, *P. aeruginosa*, *C. albicans*, and *E. coli* were separated into T^800^ + V^84^, T^350^ + V^34^, T^450^ + V^48^, T^140^ + V^18^, T^210^ + V^29^, T^270^ + V^31^, T^140^ + V^12^, and T^93^ + V^10^ correspondingly.

### 2.3. Tenfold Cross-Validation

The training/testing data set is utilized for model development through Mmachine learning techniques (MLTs) and the performance of MLTs on data was cross-validated by employing the *n*-fold cross-validation method [28]. In the current study, we used a 10-fold cross-validation (*n* = 10) method [29]. In this method, the complete data set is divided into 10 sets, from which 9 sets are concatenated (training set), and the remaining 1 is a testing set. The performance of the training set is evaluated using a testing set, and this procedure is iterated 10 times till all of the 10 sets become a testing set. Finally, the performance of all the 10 sets is averaged out for mean accuracy. Apart from internal cross-validation (training/testing) during model development, an external authentication was also performed by exploiting an independent validation dataset which was not used anywhere in training/testing.

### 2.4. Support Vector Machine

The support vector machine (SVM) is a supervised MLT which can be implemented on classification and regression data. It is based on constructing decision planes in multidimensional space that separate two classes of data. The decision planes can be linear or nonlinear. The effectiveness of SVM is based on kernel selection for efficient optimization. Some commonly used kernels are linear, polynomial (homogeneous or inhomogeneous), gaussian radial basis function, hyperbolic tangent, etc. SVM*^light^* is implemented in the development of various algorithms [29,30,31].

### 2.5. Random Forest

The random forest is an ensemble machine learning approach which operates by constructing decision trees from a training dataset. The output results from the mean prediction of individual trees for regression problems. The random forest has been implemented previously in various algorithms such as anti-flavi [32], QSPpred [29], anti-Corona [33], etc.

### 2.6. Data Preprocessing

The preprocessing of the data was performed by converting the chemical SMILES into the 3D SDF using Open Babel software because, when calculating 3D descriptors, the 3D SDF format is important [34]. The initial SMILES were extracted from the aBiofilm database. Furthermore, the command line obabel software was employed for the conversion of SMILES to 3D SDF format in batch mode. Later on, this 3D SDF was used for PaDEL molecular descriptor calculation.

### 2.7. Descriptors Calculation

Descriptors are the numerical exemplification of chemical information encoded within a symbolic representation of a molecule [27]. For the study, molecular descriptors of various dimensionality, namely 1D, 2D, and 3D, were extracted, along with the fingerprints [27]. We employed PaDEL, a molecular descriptor computing software for converting chemical structure information into fixed-length numeric vectors. It includes 16,383 dimensionality descriptors and fingerprints.

### 2.8. Features Selection

Features selection allows the selection of a subset of features that are relevant for model development. Feature selection is an important step in simplifying models, decreasing training time, reducing overfitting, etc. We used “Remove Useless” for preprocessing, followed by attribute evaluator “CfsSubsetEval” and search method “BestFirst” from the Waikato Environment for Knowledge Analysis (WEKA) package [35], to fetch out the most contributing features [27].

### 2.9. Chemical Analysis

We performed analysis of the biofilm inhibitors using Scaffold Hunter software [36]. All the biofilm inhibitors were visualized through scaffold trees, tree maps, and scaffold clouds to check their diversity. A scaffold tree allows the user to have an overview of the structure classification hierarchy and distribution of the structure in a particular database. Tree map gives the complementary space-filling representation to the established scaffold tree view of all the biofilm inhibitors on the basis of scaffolds and inhibition efficacies. The scaffold cloud provides a compact and summarized view of all the molecules in the database. We plotted the scaffold cloud using the “Ertl” layout algorithm and “EUCLIDE” distance matrix [37].

### 2.10. Performance Measures

For regression (quantitative) mode, the correlation between two variables is measured using Pearson’s correlation coefficient (PCC or R). In bioinformatics, the two variables are actual and predicted values. The range of PCC varies from −1 to +1. If PCC is −1, it indicates that observed and actual values are negatively correlated, 0 shows random prediction, while +1 displayed the positive correlation among them. PCC is calculated using the formula:

(1) $$R=\frac{n\sum_{n=1}^{n}E_{i}^{act}E_{i}^{pred}-\sum_{n=1}^{n}E_{i}^{act}\;\sum_{n=1}^{n}E_{i}^{pred}\;}{\sqrt{n\sum_{n=1}^{n}{\left(E_{i}^{act}\right)}^{2}-{\left(\sum_{n=1}^{n}E_{i}^{act}\right)}^{2}}-\sqrt{n\sum_{n=1}^{n}{\left(E_{i}^{pred}\right)}^{2}-{\left(\sum_{n=1}^{n}E_{i}^{pred}\right)}^{2}}}\;$$

where *n*, $E_{i}^{pred}$ and $E_{i}^{act}$ are the size of the test set, predicted and actual efficiencies of biofilm inhibition respectively.

The coefficient of determination (R^2^) is the statistical measure for determining the efficiency of the regression line to estimate the real data. The R^2^ varies from 0 to 1; if it is near 1, the estimated rate of regression is perfect, whereas 0 means imperfect estimation.

Mean absolute error (MAE) is the difference between actual and predicted values.

(2) $$MAE=\frac{1}{n}\overset{n}{\underset{n=1}{\sum }}\left|E_{i}^{pred}-E_{i}^{act}\right|\;$$

where, $E_{i}^{pred}$, $E_{i}^{act}$ and |$E_{i}^{pred}\;$−$\;E_{i}^{act}$| are the predicted and actual efficiencies of biofilm inhibition and absolute error. The negative values of MAE are preferred for better prediction quality.

Root mean square error (*RMSE*) is the scoring rule to measure the average magnitude of the error. Its negative values showed the efficiency of good prediction.

(3) $$RMSE=\sqrt{\frac{1}{n}\overset{n}{\underset{n=1}{\sum }}{\left(E_{i}^{pred}-E_{i}^{act}\right)}^{2}}\;$$

### 2.11. Webserver

All the prediction models were incorporated in the form of the “Biofilm-*i*” webserver (https://bioinfo.imtech.res.in/manojk/biofilmi/, 16 July 2022). The webserver is constructed using an apache server and hosted on the Linux operating system. The back end of the server is optimized using Python and Perl. The front end of the server was developed using PHP, Javascript, CSS, and HTML.

## 3. Results

We used the support vector machine technique to develop recursive regression models for generalized chemicals, group-specific (Gram-positive, Gram-negative bacteria, and fungus) and species-specific (*Pseudomonas aeruginosa*, *Staphylococcus aureus*, *Candida albicans,* and *Escherichia coli*). Moreover, we also performed chemical analyses to explore the interrelationship between chemical structure and inhibition efficacies.

### 3.1. Performance of Quantitative Structure—Activity Relationship (QSAR) Based Models Using Support Vector Machine

All the sequences of chemicals were used for feature selection by PaDel software, which resulted in 16,383 descriptors. Further, the feature selection resulted in 265, 177, 387, 111, 81, 90, 76, and 52 features among overall chemicals, Gram-positive bacteria, Gram-negative bacteria, fungus, *P. aeruginosa*, *S. aureus*, *C. albicans,* and *E. coli* respectively. 

From training/testing data sets, the Pearson’s correlation coefficient (PCC) of overall chemicals, Gram-positive bacteria, Gram-negative bacteria, fungus, *P. aeruginosa*, *S. aureus*, *C. albicans,* and *E. coli* were 0.60, 0.77, 0.62, 0.77, 0.73, 0.83, 0.70, and 0.71 respectively. Furthermore, all the models were tested using independent/validation data sets, which resulted in PCC of 0.53, 0.76, 0.60, 0.71, 0.78, 0.86, 0.82, and 0.82 correspondingly in all the above-mentioned categories. Detailed results are tabulated in Table 1.

### 3.2. Performance of Quantitative Structure–Activity Relationship (QSAR) Based Models Using Random Forest

We employed the Random Forest machine learning technique against eight predictors like overall chemicals, Gram-positive bacteria, Gram-negative bacteria, Fungus/Yeast, *P. aeruginosa*, *S. aureus*, *C. albicans* and *E. coli* with PCC of 0.52, 0.68, 0.57, 0.65, 0.65, 0.80, 0.63, 0.63 respectively. However, the independent datasets performed equally well as shown in Supplementary Table S1.

### 3.3. Analyses

**Figure 1:**
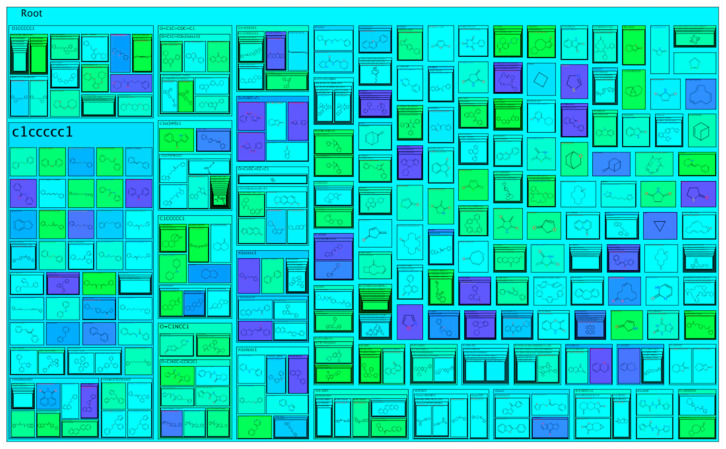
Tree map view of 884 experimentally validated biofilm inhibitors where the value of ECFP4 fingerprint is shown in different colors (yellow color depicts lowest and green color displays highest value).

Three types of analyses were performed, and overall biofilm inhibitors were presented in the form of scaffold tree, tree map, and scaffold cloud. The scaffold tree results in diverse branches with a combination of singlet and multiplex branches. The most cluttered branch has a backbone of benzene with 159 molecules, followed by pyridine, tertrahydropyran, azetidinone and pyran-4-one with 22, 21, 14, and 3 different chemicals respectively. Furthermore, the tree map view (Figure 1) depicts a more detailed view of the correlation between scaffold and biofilm inhibition efficiency. The scaffold of a benzene ring was available in 260 chemicals with the majority showing inhibition efficacy between 10 and 50%, the azetidinone backbone was available in 13 chemicals, showing an inhibition efficiency with most chemicals above 60%, and the pyran-4-one was available in 31 compounds, possessing an inhibition efficiency of 30–100% in the majority of cases.

Moreover, the molecular cloud view (Figure S1) represents a brief and compact view of all the experimentally validated biofilm inhibitors on the basis of their distribution and inhibition. It displayed that the scaffolds of benzene, pyridine, tertrahydropyran, azetidinone, and pyran-4-one, are available in most of the biofilm inhibitors and possess an average inhibition efficacy of 50%.

### 3.4. Web Server

**Figure 2:**
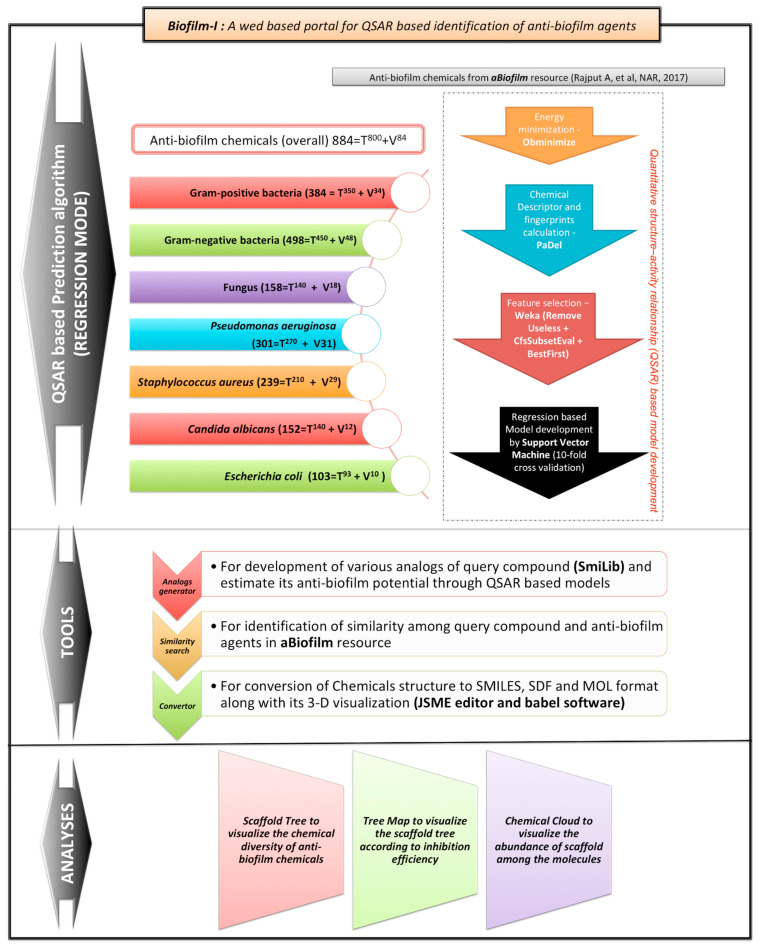
The overall architecture of the biofilm-*i* web portal. The overall architecture of the biofilm-i web portal. The data was taken from aBiofilm resource [15].

All the predictors and analysis tools were integrated into the form of an open-access web portal named Biofilm-*i* (https://bioinfo.imtech.res.in/manojk/biofilmi/, 16 July 2022). It contains eight predictors, three tools, and data visualization modules. The overall architecture of the biofilm-*i* is provided in Figure 2.

*Predictors:* The Biofilm-*i* web portal contains eight algorithms for predicting generalized chemicals, Gram-positive bacteria, Gram-negative bacteria, fungus, *P. aeruginosa*, *S. aureus*, *C. albicans,* and *E. coli*. The input can be provided in (multi) SDF format. The job ID would be assigned to every query for checking the job status and retrieving the results. The user can wait until the completion of the job or can use our “Check Job Status” facility provided in the “Predictor” menu for fetching the results. The input–output of the generalized predictor is provided in Figure S2. The results are displayed in a tabulated format including query ID provided by the user, converted simplified molecular-input line-entry system (SMILES), biofilm inhibition efficiency, important drug-like properties, and similarity search in the aBiofilm resource.

*Tools*: The biofilm-*i* web server comprises three tools i.e., conversion, similarity, and analog generator. The “conversion” tools aid the user(s) to draw the chemical and retrieve the output as SMILES, SDF, and mol format along with the 3-D view of the query chemical. Furthermore, the user can use the SDF file as input in any of the predictor(s). The “similarity” tool helps the user to scan the aBiofilm database and retrieve the similar chemical(s) with a query. However, the “analog generator” tool provides the facility to the user(s) to generate the analogs of the provided scaffold, building blocks, and linkers. The designed analogs can be predicted for biofilm inhibition potential in any of the eight algorithms i.e., generalized chemicals, Gram-positive bacteria, Gram-negative bacteria, fungus, *P. aeruginosa*, *S. aureus*, *C. albicans,* and *E. coli*.

## 4. Discussion

Biofilms are the most robust colonization form of microbes and showed up to 1000-fold resistance to antibiotics [38]. It encompasses a highly specialized form of approaches to fight against environmental cues, including antibiotics, such as an expression of efflux pumps, polysaccharide enriched matrix, oxygen gradients, and many more [39,40,41]. Hence, it is important to target biofilms to overcome the menace of antibiotic resistance globally [42]. Therefore, we developed a web-based platform named “Biofilm-*i*” for predicting the potential of (un)known chemicals to degrade biofilms. It also encompasses various analysis tools for exploring the query compounds.

Biofilm-*i* is the first regression-based prediction algorithm that possesses the ability to identify the biofilm inhibition efficacy of chemicals (generalized group-specific and species-specific) on a single platform. However, we also developed a tool integrated into the aBiofilm resource for predicting the biofilm inhibition potential in classification mode (qualitative), i.e., low and high [15]. Only one chemical can be predicted at a time by the predictor tool in aBiofilm. Contrary to that, our present web portal is typically quantitative and possesses a facility for predicting multiple chemicals in batch mode. Moreover, it incorporates various analysis tools to explore the query chemical(s) in more detail such as scanning for similar compounds in the comprehensive aBiofilm resource, fetching different chemical formats by merely drawing on the canvas of JSME editor, and designing the analogs of the query chemicals and predicting their biofilm inhibition efficiency. High-performance models are integrated into the webserver for predicting the (un)known chemical in the Biofilm-*i*.

We used a 10-fold cross-validation approach for all the models developed through the support vector machine technique. We utilized 2D, 3D descriptors, and fingerprints for the development of models so as to harbor all the topological and geometric properties of chemicals. Among all the models, the performance of the species-specific predictor was better than the group-specific and generalized predictors because a specific type of chemical is active against a particular group of microbes. The over-optimization issue during the model development was managed by taking only the relevant and most contributing features rather than all features. The internal, as well as external, validation of the models was carried out during training/testing and independent validation data sets. Both the validation methods performed almost equally well. Therefore, all the developed models are very robust in all aspects and have the ability to predict the percentage inhibition efficiency of (un) known chemicals with high accuracy.

Despite the predictors, we are providing the facility to the users to perform various analyses on their data. For example, through the analog design option, users can design various analogs of the query molecule(s), predict the inhibition potential, and then fetch the most active biofilm degrading analog, rather than the original chemical. Furthermore, users can check for similar compounds (if available) in the aBiofilm repository, which are already experimentally validated against specific microbial biofilms. To make the web server more user friendly, we incorporated a format conversion facility for the chemicals. Moreover, we explored all the experimentally validated biofilm inhibiting chemicals and tried to correlate their common scaffold and reported biofilm inhibition efficacy. We concluded that chemicals having scaffolds of cyclic or aromatic rings such as benzene, pyridine, tertrahydropyran, azetidinone, and pyran-4-one, are more preferred than aliphatic chains and possess high inhibition potential. Therefore, researchers can focus on developing efficacious inhibitors enriched with cyclic or aromatic rings.

There are a few software packages available for predicting the biofilm inhibition efficacy of peptides and chemicals e.g., dPABBs [21], BIPEP [23], aBiofilm predictor [15], and Molib [24]. However, they are developed using classification-based approaches and some use publicly available data from various repositories. For the first time, we are using a regression-based approach to the experimentally validated data of the percentage inhibition of biofilm inhibition chemicals which is named ‘Biofilm-*i*’ (https://bioinfo.imtech.res.in/manojk/biofilmi/, 16 July 2022). Moreover, the current study is developed for overall generalized chemicals, as well as for specific species, e.g., *Staphylococcus aureus* (Gram-positive bacteria), *Pseudomonas aeruginosa* (Gram-negative bacteria), *Candida albicans* (fungus or yeast), and *Escherichia coli* (Gram-negative bacteria).

Biofilm inhibitors can disrupt biofilms and also enhance conventional antibiotics through synergistic effects similar to that of adjuvants increasing the efficacy of vaccines. They have demonstrated even greater promise by killing multidrug-resistant strains, including ESKAPE pathogens [43]. Researchers have been working hard to develop various biofilm inhibitors for the last three decades due to their immense therapeutic potential. However, computational resources in this important field are lacking. In this regard, the Biofilm-*i* prediction algorithm would be of tremendous help to researchers in developing novel biofilm inhibitors speedily and effectively. It would reduce the time spent and cost of experimental biologists screening a large library of compounds. Researchers can use our web resource to initially filter out the highly efficient compounds from the library rather than experimentally screen them. They can also in-silico design and predict the compounds and their respective analogs. We hope that our Biofilm-*i* web portal will be a one-stop solution to the problem of designing novel and efficient biofilm inhibitors. It would prove to be a powerful computational tool for the scientific community to curb the problem of antibiotic resistance.

## Figures and Tables

**Table 1:** Performance of all the eight predictors (both training/testing and independent validation) using a regression-based approach developed using the support vector machine method, along with the final descriptors employed individually.

Models Used	Data Sets	Features	Pearson’s Correlation Coefficient
Chemicals (Overall)	Training/Testing data set (T^800^)	265	0.60
	Independent Validation data set (V^84^)		0.53
Gram-positive bacteria	Training/Testing data set (T^350^)	177	0.77
	Independent Validation data set (V^34^)		0.76
Gram-negative bacteria	Training/Testing data set (T^450^)	387	0.62
	Independent Validation data set (V^48^)		0.60
Fungus/Yeast	Training/Testing data set (T^140^)	111	0.77
	Independent Validation data set (V^18^)		0.71
*Pseudomonas aeruginosa*	Training/Testing data set (T^270^)	81	0.73
	Independent Validation data set (V^31^)		0.78
*Staphylococcus aureus*	Training/Testing data set (T^210^)	90	0.83
	Independent Validation data set (V^29^)		0.86
*Candida albicans*	Training/Testing data set (T^140^)	76	0.70
	Independent Validation data set (V^12^)		0.82
*Escherichia coli*	Training/Testing data set (T^93^)	52	0.71
	Independent Validation data set (V^10^)		0.82

## Data Availability

Not applicable.

## Supplementary Materials

The following supporting information can be downloaded at: https://www.mdpi.com/article/10.3390/molecules27154861/s1, Figure S1. Scaffold cloud view of 884 experimentally validated biofilm inhibitors where biofilm inhibition efficiency shown in colors (blue color depicts 0% and green color displays 100%). Figure S2. Input output of generalised predictor available in biofilm-*i* web portal. Table S1. Performance of all the eight predictors (both training/testing and independent validation) using regression based approach developed using Random Forest along with the final descriptors employed individually.
