# Supplementary material for: Biofilm-i: A Platform for Predicting Biofilm Inhibitors Using Quantitative Structure—Relationship (QSAR) Based Regression Models to Curb Antibiotic Resistance
Source: Molecules. 2022 Jul 29;27(15):4861. doi: 10.3390/molecules27154861 (PMC9369795; doi:10.3390/molecules27154861)
Supplement: Supplementary file 1 [file molecules-27-04861-s001.zip › molecules-1781918-supplementary.pdf]

# Biofilm-i: A platform for predicting biofilm inhibitors using Quantitative structure–activity relationship (QSAR) based regression models to curb antibiotic resistance

**Akanksha Rajput[1], Kailash T. Bhamare[1,2], Anamika Thakur[1,2] and Manoj Kumar[1,2*]**

[1]Virology Unit and Bioinformatics Centre, Institute of Microbial Technology, Council of Scientific and Industrial Research (CSIR), Sector 39-A, Chandigarh-160036, India

[2]Academy of Scientific and Innovative Research (AcSIR), Ghaziabad-201002, India

*** Correspondence:**
Corresponding Author
manojk@imtech.res.in

**Supplementary information**

**Figure S1.** Scaffold cloud view of 884 experimentally validated biofilm inhibitors where biofilm inhibition efficiency shown in colors (blue color depicts 0% and green color displays 100%)

**Figure S2.** Input output of generalised predictor available in biofilm-i web portal

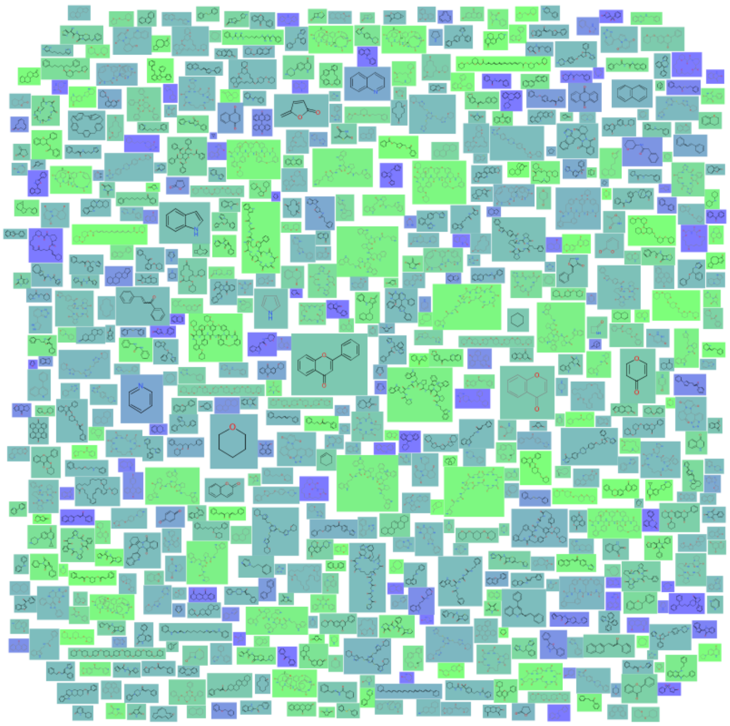

**Figure S1.** Scaffold cloud view of 884 experimentally validated biofilm inhibitors where biofilm inhibition efficiency shown in colors (blue color depicts 0% and green color displays 100%)

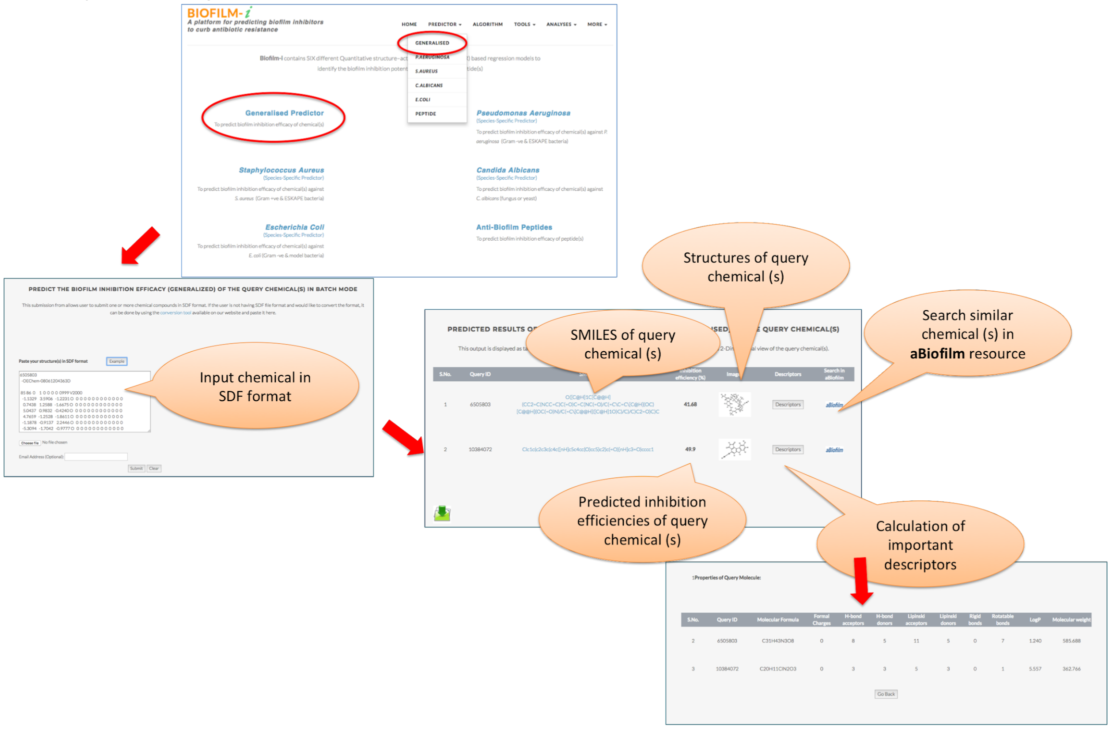


**Figure S2.** Input output of generalised predictor available in biofilm-i web portal

**Supplementary Table**

**Supplementary Table S1.** Performance of all the eight predictors (both training/testing and independent validation) using regression based approach developed using Random Forest along with the final descriptors employed individually

| Model used | Data Sets | Features | Pr-cor | RMSE | MAE |
|---|---|---|---|---|---|
| Chemicals (Overall) | Training/Testing data set ($T^{800}$) | 265 | 0.52 | 24.22 | 18.46 |
| | Independent Validation data set ($V^{84}$) | | 0.45 | 27.17 | 21.12 |
| | | | | | |
| Gram-positive bacteria | Training/Testing data set ($T^{350}$) | 177 | 0.68 | 22.03 | 16.11 |
| | Independent Validation data set ($V^{34}$) | | 0.67 | 22.49 | 16.65 |
| | | | | | |
| Gram-negative bacteria | Training/Testing data set ($T^{450}$) | 387 | 0.57 | 23.95 | 18.87 |
| | Independent Validation data set ($V^{48}$) | | 0.53 | 22.03 | 16.26 |
| | | | | | |
| Fungus/Yeast | Training/Testing data set ($T^{140}$) | 111 | 0.65 | 19.10 | 12.55 |
| | Independent Validation data set ($V^{18}$) | | 0.53 | 17.33 | 11.57 |
| | | | | | |
| *Pseudomonas aeruginosa* | Training/Testing data set ($T^{270}$) | 81 | 0.65 | 21.79 | 16.51 |
| | Independent Validation data set ($V^{31}$) | | 0.62 | 22.46 | 16.27 |
| | | | | | |
| *Staphylococcus aureus* | Training/Testing data set ($T^{210}$) | 90 | 0.80 | 19.36 | 14.02 |
| | Independent Validation data set ($V^{29}$) | | 0.78 | 16.74 | 12.15 |
| | | | | | |
| *Candida albicans* | Training/Testing data set ($T^{140}$) | 76 | 0.63 | 19.15 | 12.83 |
| | Independent Validation data set ($V^{12}$) | | 0.79 | 15.06 | 8.88 |

| | | | | | |
|---|---|---|---|---|---|
| *Escherichia coli* | Training/Testing data set ($T^{93}$) | 52 | 0.63 | 22.56 | 17.59 |
| | Independent Validation data set ($V^{10}$) | | 0.86 | 17.40 | 13.59 |
